# Genomic insights into virulence factors affecting tissue-invasive *Klebsiella pneumoniae* infection

**DOI:** 10.1186/s12941-022-00494-7

**Published:** 2022-02-05

**Authors:** Takashi Matono, Masatomo Morita, Nodoka Nakao, Yuji Teshima, Makoto Ohnishi

**Affiliations:** 1 Department of Infectious Diseases, Aso Iizuka Hospital, 3-83 Yoshio, Iizuka, Fukuoka 820-8505 Japan; 2 Department of Bacteriology I, National Institute of Infectious Diseases, Tokyo, Japan; 3 Department of Clinical Laboratory, Aso Iizuka Hospital, Fukuoka, Japan

**Keywords:** Hypervirulent *Klebsiella pneumoniae*, Virulence factor, Aerobactin, *rmpA*

## Abstract

**Background:**

The key virulence factors responsible for hypervirulent *Klebsiella pneumoniae* (hvKp) infection remains elusive.

**Methods:**

We analyzed *K. pneumoniae* isolates collected between 2017 and 2019 and defined hvKp as a pyogenic infection. Classical *K. pneumoniae* (cKp) involved a non-invasive infection or uncomplicated bacteremia. Isolates belonging to the *K. pneumoniae* species complex were excluded.

**Results:**

We analyzed 112 isolates, including 19 hvKp, 67 cKp, and 26 colonizers, using whole-genome sequencing. Population genomics revealed that the K1-sequence type (ST) 82 (O1v1) clade was distinct from that of the K1-ST23 (O1v2) clone. The virulence gene profiles also differed between K1-ST82 (aerobactin and *rmpA*) and K1-ST23 (aerobactin, yersiniabactin, salmochelin, colibactin, and *rmpA*/*rmpA2*). The K2 genotype was more diverse than that of K1. A neighboring subclade of K1-ST23 (comprising ST29, ST412, ST36, and ST268) showed multidrug resistance and hypervirulence potentials. Logistic-regression analysis revealed that diabetes mellitus was associated with *K. pneumoniae* infection (odds ratio [OR]: 4.11; 95% confidence interval [CI]: 1.14–14.8). No significant association was found between hvKp diagnosis and clinical characteristics, such as diabetes mellitus or community acquisition. However, the K1 genotype (OR: 9.02; 95% CI: 2.49–32.7; positive-likelihood ratio [LR]: 4.08), *rmpA* (OR: 8.26; 95% CI: 1.77–38.5; positive LR: 5.83), and aerobactin (OR: 4.59; 95% CI: 1.22–17.2; positive LR: 3.49) were substantial diagnostic predictors of hvKp.

**Conclusions:**

The K1 genotype, *rmpA*, and aerobactin are prominent predictors of hvKp, suggesting that further pyogenic (metastatic) infection should be examined clinically. These findings may shed light on key hvKp virulence factors.

**Supplementary Information:**

The online version contains supplementary material available at 10.1186/s12941-022-00494-7.

## Introduction

Severe community-acquired metastatic *Klebsiella pneumoniae* infection was first reported in 1986 in Taiwan [1]. Hypervirulent *K. pneumoniae* (hvKp) causes life-threatening infections such as endophthalmitis, liver abscess, meningitis, and necrotizing soft tissue infection, with clinical features that differ from nosocomial classical *K. pneumoniae* (cKp) infections. Although capsular serotypes (K1/K2) and hypermucoviscosity (positive string test) have been traditionally considered as virulence factors suggestive of hvKp, siderophores and regulator of mucoid phenotype A (*rmpA*)/*rmpA2* were recently identified as prominent virulence factors [2, 3]. Siderophores (including enterobactin, yersiniabactin, aerobactin, and salmochelin) play roles in iron uptake into bacteria, leading to enhanced growth. Likewise, *rmpA*/*rmpA2* increase the functional advantage of serum resistance observed with hypermucoviscosity. Recently, virulence plasmids, such as pK2044 and pLVPK (encoding the aerobactin, salmochelin, and *rmpA* genes) have also been noted [4, 5].

The geographic distributions of hvKp and cKp differ; hvKp is endemic in the Asian Pacific Rim, and antimicrobial-resistant cKp is increasingly emerging in western countries [4, 5]. Japan is one of the hvKp-endemic areas, resulting in an alarming issue in actual clinical settings [6, 7]. Occasionally, nosocomial- and healthcare-associated hvKp infections occur [6], which render the classical predictor for hvKp (community-acquisition) not meaningful. In such hvKp-endemic settings, well-designed clinical studies of the predictors of hvKp infection are relatively limited to date. Hence, the aim of this study was to evaluate the factors associated with progression from colonization to infection and identify the virulence factors associated with developing hvKp infection by using whole-genome sequencing.

## Materials and methods

### Study design and setting

This retrospective observational study was designed to assess clinical and molecular virulence factors in patients with *K. pneumoniae*. This study was conducted at the Aso Iizuka Hospital (AIH), a tertiary care hospital in Fukuoka, Japan, with 1048 inpatient beds and an adult/neonatal intensive care unit (ICU). The clinical and microbiological data used for this study were obtained from a microbiological laboratory and by reviewing charts at the AIH.

This study was approved by the Institutional Review Board at AIH (approval number 17182) and conducted according to the principles of the Declaration of Helsinki. The need for informed consent was waived because only data collected during clinical practice were used in this study.

### Bacterial isolates and the study population

**Fig. 1:**
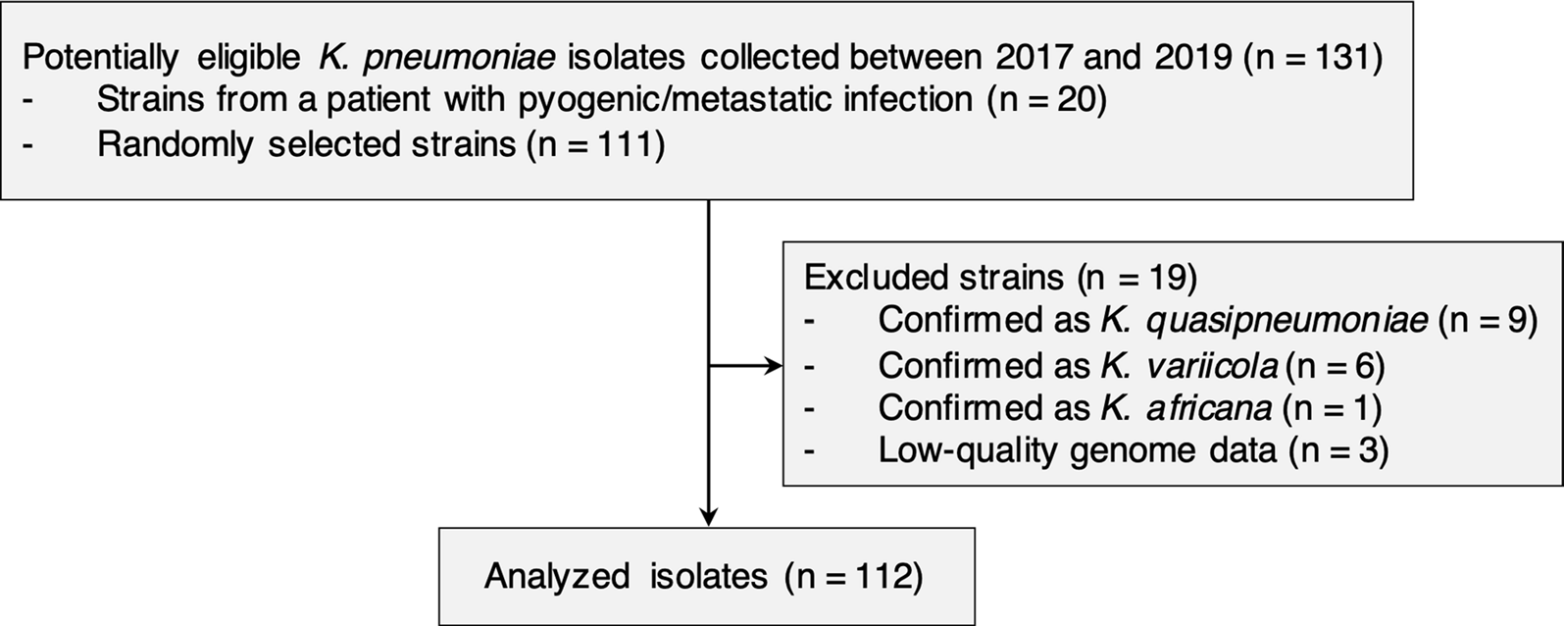
Flow diagram showing the selection criteria for *K. pneumoniae* analysis. *K. quasipneumoniae*, *K. variicola*, and *K. africana* were identified using whole-genome sequencing and Kleborate

*K. pneumoniae* isolates were identified by matrix-assisted laser desorption/ionization–time-of-flight mass spectrometry (MALDI–TOF MS) using a MALDI Biotyper (Bruker Daltonics, Kanagawa, Japan) and stored at the AIH. We included all potentially eligible *K. pneumoniae* isolates recovered from a patient with pyogenic/metastatic infection at the AIH between April 2017 and April 2019 (Fig. 1). Additionally, we included randomly selected *K. pneumoniae* isolates from patients with infected and carriage statuses during the same period, as controls. Isolates recovered from a previously enrolled patient were excluded. We also excluded isolates that are members of the *K. pneumoniae* species complex, such as *K. quasipneumoniae* and *K. variicola*, identified using whole-genome sequencing. Of the 131 potentially eligible isolates, 19 were excluded and 112 *K. pneumoniae* isolates were analyzed.

### Measurements and definitions

We extracted patient characteristics including age, sex, underlying diseases/conditions, site of infection, ICU admission, and in-hospital death. We used the Charlson index to assess comorbidity and mortality [8]. Community-acquired infection was defined as a strain identified at the outpatient clinic or within 48 h after admission. HvKp, cKp, and colonization were classified based on clinical presentation at the time of isolation. We defined hvKp as a pyogenic infection where *K. pneumoniae* was isolated from a sterile site (except for blood), such as deep-seated tissue, abscess, and cerebrospinal, intraocular, pleural, pericardial, or joint fluids. We defined cKp as a non-invasive infection or uncomplicated bacteremia, caused by cholecystitis, cholangitis, and pyelonephritis. We defined colonization as a carriage status that was not causative bacteria. A positive string test was defined as a > 5 mm viscous string in the colony. The breakpoints of antibiotics were based on Clinical Laboratory Standard Institute document M100-S29. The multidrug resistant (MDR) strains identified included extended-spectrum beta-lactamase (ESBL)-producing strains, AmpC beta-lactamase-producing strains, and carbapenem-resistant strains.

### Whole-genome sequencing and phylogenetic analyses

Genomic DNA was prepared using a DNeasy Blood & Tissue Kit (Qiagen, Hilden, Germany) at the AIH. Further genomic analysis was conducted at the Department of Bacteriology I at the National Institute of Infectious Diseases in Tokyo, Japan. Genomic DNA libraries were prepared using a Nextera XT DNA Sample Prep Kit (Illumina, San Diego, CA, USA). Paired-end (300 × 2 bp) short reads for each library were sequenced on a MiSeq instrument (Illumina). Sufficient DNA sequence reads were generated for at least 40-fold depth of the reference genome (described below). Genome assembly was performed using SPAdes software, version 3.13.1 with default parameters [9]. Variants were called using Snippy software, version 4.3.6 (https://github.com/tseemann/snippy) with the *K. pneumoniae* subsp. *pneumoniae* NTUH-K2044 genome (GenBank accession number: AP006725) as a reference. We excluded single-nucleotide variations (SNVs) in recombinogenic regions detected using the Gubbins software, version 2.3.4 [10], along with SNVs in the repetitive NTUH-K2044 genome regions, which were identified using the NUCmer program for studying core genome phylogeny [11]. The remaining 13,582 SNVs were concatenated to generate a pseudosequence for phylogenetic analysis; maximum-likelihood phylogenetic analysis was performed using IQ-TREE software with 1,000 ultrafast bootstrap replicates [12]. The taxonomy of *K. pneumonia* species, sequence type (ST), capsular genotype (K locus), lipopolysaccharide genotype (O locus), *rmpA*, *rmpA2*, and genes encoding aerobactin, yersiniabactin, salmochelin, and colibactin in the draft genome were identified using Kleborate (https://github.com/katholt/Kleborate) [13, 14]. The nucleotide-sequence data were deposited in the DNA Data Bank of Japan Sequenced Read Archive under accession numbers DRX270567–270,695 (Additional file 1: Table S1) [15].

### Statistical analysis

Patient and microbiological characteristics were compared between infection and colonization, and hvKp and cKp. The chi-square test or Fisher’s exact test was used for nominal variables, and the Mann–Whitney U test was used for continuous variables. Logistic-regression analysis was performed to predict potential risk factors for infection and hvKp, based on odds ratios (ORs) and 95% confidence intervals (CIs). The diagnostic accuracy of the microbiological profiles for hvKp, including the sensitivity, specificity, positive/negative predictive value, or positive/negative likelihood ratio (LR), were calculated using 2 × 2 tables, and the 95% CIs were calculated using MedCalc for Windows, version 16.2 (MedCalc Software, Ostend, Belgium). Statistical significance was defined as a two-tailed p-value of < 0.05, using the 95% CI. All analyses were performed using SPSS for Windows version 21 (IBM Corp., Armonk, NY, USA).

## Results

### Genomic epidemiology of analyzed *K. pneumoniae* isolates

Of the 112 *K. pneumoniae* isolates analyzed, 19 were hvKp, 67 were cKp (including 55 non-invasive infections and 12 cases of uncomplicated bacteremia), and 26 were colonizers. The 16 strains that were excluded were confirmed as members of the *K. pneumoniae* species complex, namely *K. variicola* subsp. *variicola* (n = 6), *K. quasipneumoniae* subsp. *similipneumoniae* (n = 7), *K. quasipneumoniae* subsp. *quasipneumoniae* (n = 2), and *K. africana* (n = 1; Additional file 2: Table S2). The rate of consistency between the string test and *rmpA* and/or *rmpA2* was 84% (94 out of 112 isolates). The inconsistent patterns included a positive string test but the absence of *rmpA*/*rmpA2* (n = 11), or a negative string test but the presence of *rmpA*/*rmpA2* (n = 7). Gene clusters inferring virulence plasmid (encoding aerobactin, salmochelin, and *rmpA* genes) were noted in 58% (11/19) of hvKp strains, 64% (43/67) of cKp strains, and 58% (15/26) of colonizers, respectively. Phylogenetic analysis of the 112 isolates revealed that a subset of 12 K1-ST23 (O1 variant 2) strains formed a specific cluster with a reference strain, in which the median pairwise SNV distance was 7 (range: 1–14; Fig. 2). A subset of five K1-ST82 (O1v1) strains clustered together; however, this clade was distinct from K1-ST23. The virulence-gene profiles were also different between K1-ST23 and K1-ST82. Namely, the K1-ST23 strains tended to harbor siderophore gene clusters (aerobactin, yersiniabactin, and salmochelin), colibactin, *rmpA*, and *rmpA2*, whereas the K1-ST82 strains harbored aerobactin and *rmpA*. Similar virulence-gene profiles (compared with that of K1-ST23) were found in K62-ST36, K20-ST268, K2-ST239, and K2-ST65; however, 7 out of 11 (63%) K20-ST268 isolates showed colonization. The K2 genotype was more diverse than the K1 genotype; the former predominantly belonged to two clustered ST65/ST375 (neighboring ST25) and ST86 clades, which were distinct from ST14 and ST2039.

**Fig. 2:**
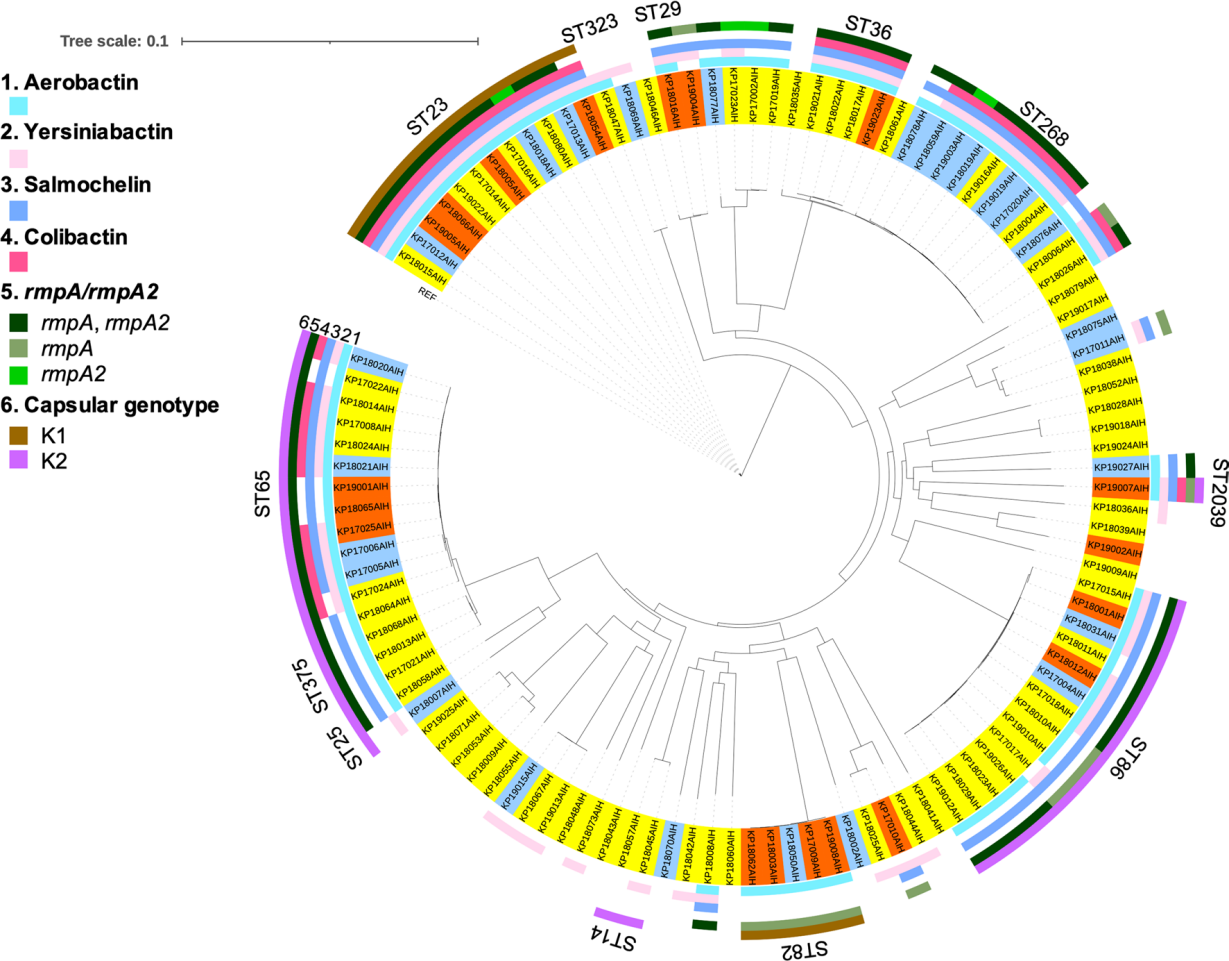
Phylogenetic distribution of genetic virulence factors in 112 *K. pneumoniae* isolates. We identified 13,582 SNVs. The highlighted strains are clinically pathogenic (orange, hypervirulent *K. pneumoniae*; yellow, classical *K. pneumoniae*; sky blue, colonization). The non-highlighted strain (NTUH-K2044) is a reference *K. pneumoniae* strain. K1-ST82 was distinct from K1-ST23 and harbored a different virulence-gene profile. The K2 genotype predominantly belongs to the ST65/ST375 (neighboring ST25) and ST86 clades, which are distinct from ST14 and ST2039. SNVs, single-nucleotide variations; ST, sequence type

Of the 112 isolates, nine (8%) MDR strains were identified, including six ESBL-producing strains, two AmpC beta-lactamase-producing strains, and one carbapenem-resistant strain. Two K21-ST323 strains, harboring only one or two virulence loci (yersiniabactin and/or aerobactin), were ESBL producers, which shared a recent common ancestor with the K1-ST23 clone. The ST36 (K102, K62, and K27) strains and K20-ST268 strains clustered together and had similar virulence profiles. However, the ST36 clade included no MDR strains, whereas four of 11 (36%) K20-ST268 strains were MDR strains (two ESBL-producing strains, one AmpC beta-lactamase-producing strains, and one carbapenem-resistant strain).

### Factors related to infection and hvKp

Among the 112 isolates (86 infections and 26 colonizers), diabetes mellitus was more frequent in patients with infection than in those with colonization (35% versus 12%, p = 0.022; Additional file 2: Table S3). Logistic-regression analysis revealed a significant association between infection and diabetes mellitus (OR: 4.11; 95% CI: 1.14–14.8), but not other microbiological variables (Additional file 2: Table S4). Of the 19 patients with hvKp, the median age was 74 years (range: 50–90 years), 74% of whom were men (Additional file 2: Table S5). Amongst all the hvKp strains, the most common capsular genotypes were K1 (n = 8, 42%; ST23 [n = 4] and ST82 [n = 4]), followed by K2 (n = 6, 32%; ST65 [n = 3], ST86 [n = 2], and ST239 [n = 1]), and K54 (n = 2, 11%; ST29); the most predominant lipopolysaccharide genotypes were O1 (n = 17, 89%; O1v2 [n = 10] and O1v1 [n = 7]), followed by O2v1 (n = 1), and O3b (n = 1). Diabetes mellitus, community-acquired infection, ICU admission, and in-hospital mortality were not significantly different between patients with hvKp or cKp (Table 1). However, hvKp infection was associated with a higher frequency of liver cirrhosis (16% versus 1%, p = 0.032), a positive string test (84% versus 57%, p = 0.029), the O1 genotype (89% versus 54%, p = 0.005), K1 genotype (42% versus 7%, p = 0.001), aerobactin (84% versus 54%, p = 0.016), and *rmpA* (89% versus 51%, p = 0.002) than that of cKp infection. Logistic-regression analysis showed a significant association between hvKp diagnosis and each predictor, including liver cirrhosis (OR: 12.4; 95% CI: 1.21–127), positive string test (OR: 4.07; 95% CI: 1.08–15.3), O1 genotype (OR: 7.32; 95% CI: 1.57–34.2), K1 genotype (OR: 9.02; 95% CI: 2.49–32.7), aerobactin (OR: 4.59; 95% CI: 1.22–17.2), and *rmpA* (OR: 8.26; 95% CI: 1.77–38.5; Table 2). The sensitivity and specificity of a positive string test for hvKp were 84.2% and 43.3%, respectively (Table 3). The highest positive LR was 5.83 (95% CI: 1.47–36.0) for *rmpA*, followed by 4.08 (95% CI: 1.74–7.48) for the K1 genotype, 3.49 (95% CI: 1.08–14.7) for aerobactin, 1.67 (95% CI: 1.14–1.91) for O1 genotype, and 1.49 (95% CI: 0.99–1.79) for a positive string test. The lowest negative LR was 0.23 (95% CI: 0.038–0.79) for O1 genotype with a negative predictive value of 93.9% (95% CI: 81.7–98.9).

**Table 1:** Clinical and microbiological characteristics of patients with hvKp or cKp (n = 86)

	hvKp (n = 19)	cKp (n = 67)	p value
Age (years), median (IQR)	74 (68–75)	78 (68–84)	0.064
Male, n (%)	14 (74)	38 (57)	0.18
Comorbidity, n (%)			
Diabetes mellitus	8 (42)	22 (33)	0.45
Malignancy	0 (0)	24 (36)	0.002
Immunocompromised conditions	2 (11)	9 (13)	0.54
Liver cirrhosis	3 (16)	1 (1)	0.032
Chronic kidney disease	2 (11)	6 (9)	0.57
Charlson index, median (IQR)	2 (1–3)	3 (2–5)	0.052
Community acquired, n (%)	11 (58)	34 (51)	0.58
Admitted to ICU, n (%)	3 (16)	8 (12)	0.46
In-hospital mortality, n (%)	6 (32)	16 (24)	0.34
Microbiological profile, n (%)			
Positive string test	16 (84)	38 (57)	0.029
O1^*^	17 (89)	36 (54)	0.005
O2^*^	1 (5)	21 (31)	0.016
O3	1 (5)	8 (12)	0.36
K1	8 (42)	5 (7)	0.001
K2	6 (32)	22 (33)	0.92
Aerobactin	16 (84)	36 (54)	0.016
Yersiniabactin	12 (63)	30 (45)	0.16
Salmochelin	14 (74)	35 (52)	0.096
Colibactin	7 (37)	16 (24)	0.26
* rmpA*	17 (89)	34 (51)	0.002
* rmpA2*	10 (53)	32 (48)	0.71

hvKp, hypervirulent *K. pneumoniae*; cKp, classical *K. pneumoniae*; IQR, interquartile range

*The three O1/O2v2 strains in cKp

**Table 2:** Variables analyzed for predicting hvKp infection

Variables	OR (95% CI)	p value
Diabetes mellitus	1.49 (0.52–4.23)	0.46
Liver cirrhosis	12.4 (1.21–127)	0.034
Community-acquired	1.34 (0.48–3.73)	0.58
Positive string test	4.07 (1.08–15.3)	0.038
O1	7.32 (1.57–34.2)	0.011
O2	0.12 (0.02–0.97)	0.047
K1	9.02 (2.49–32.7)	0.001
K2	0.94 (0.32–2.82)	0.92
Aerobactin	4.59 (1.22–17.2)	0.024
Yersiniabactin	2.11 (0.74–6.04)	0.16
Salmochelin	2.56 (0.83–7.91)	0.11
Colibactin	1.86 (0.63–5.52)	0.26
*rmpA*	8.26 (1.77–38.5)	0.007
*rmpA2*	1.26 (0.44–3.37)	0.71

hvKp, hypervirulent *K. pneumoniae*; OR, odds ratio; CI, confidence interval

**Table 3:** Microbiological diagnostic predictive values for hvKp

Characteristics	Sensitivity (%)	Specificity (%)	PPV (%)	NPV (%)	LR+	LR–
Positive string test	84.2	43.3	29.6	90.6	1.49	0.37
O1	89.5	46.3	32.1	93.9	1.67	0.23
K1	61.5	84.9	42.1	92.5	4.08	0.45
Aerobactin	30.8	91.2	84.2	46.3	3.49	0.76
*rmpA*	33.3	94.3	89.5	49.3	5.83	0.71

hvKp, hypervirulent *K. pneumoniae*; PPV, positive predictive value; NPV, negative predictive value; LR, likelihood ratio

## Discussion

In this study, we assessed the *K. pneumoniae*-population structure by focusing on virulence-gene profiles and evaluating clinical and microbiological factors related to infection or hvKp in patients with *K. pneumoniae*. We found that the virulence-gene profiles differed between the K1-ST23 clone and the distinct K1-ST82 cluster. We identified diabetes mellitus as a risk factor for developing *K. pneumoniae* infection (OR: 4.11; 95% CI: 1.14–14·8). In addition, *rmpA* (positive LR, 5.83), the K1 genotype (positive LR, 4.08), and aerobactin (positive LR 3.49) were substantial predictors of hvKp. These findings may shed light on the unknowns to date, and potential utility of clinically assessing virulence factors in the future.

The present study had three important findings. First, our study appropriately dealt with the occasionally misleading *K. pneumonia* taxonomy and definition of hvKp. Members of the *K. pneumoniae* species complex, such as *K. quasipneumoniae* and *K. variicola*, have been misclassified as *K. pneumoniae* using conventional biochemical methods and even MALDI–TOF MS in clinical laboratories [4, 6], which can lead to misinterpretation of the study findings. The *K. pneumoniae* species complex, which comprises 10%–20% of clinical isolates identified as *K. pneumoniae* [4], occasionally has a positive string test and siderophores [6, 16], and can rarely cause liver abscess [17, 18]. Therefore, our study excluded the misidentification of 16 of 128 (13%) *K. pneumoniae* species complex isolates by whole-genome sequencing to reduce the potential bias. Furthermore, different definitions of hvKp have been used in the literatures, which can lead to confusion for readers and researchers alike, for example, hypermucoviscous *K. pneumoniae* [19, 20]; the presence of *rmpA*, *rmpA2*, salmochelin, and aerobactin [6]; invasive infection including uncomplicated bacteremia [3]; and tissue-invasive infection [2]. Based on our clinical question, we defined hvKp as a pyogenic infection to explore the risk factors for complicated (metastatic or pyogenic) infection that requires further examination in a clinical setting. Previously, hvKp infection was considered to be related to community acquisition [3, 5]; however, hvKp was not associated with community-acquired infection in our study. This finding may be because the prevalence of nosocomial and healthcare-associated hvKp has increased in the hvKp-endemic Asian Pacific Rim [6, 21]. As seen in our results, clinical characteristics such as diabetes mellitus or community acquisition may not be useful as predictors of hvKp infection in hvKp-rich settings. Thus, we believe that our study method, including the identification of strains and the specific definition of hvKp, enabled us to obtain results of practical significance in actual clinical practice. Our findings suggest that if the K1 genotype, *rmpA*, and aerobactin are present in a collected *K. pneumoniae* isolate, further systemic examination should be performed to determine pyogenic (metastatic) infection, clinically.

Second, our study demonstrates that several unknown aspects other than hypervirulence plasmids still exist, particularly regarding the hypermucoviscous phenotype and factor(s) for developing hvKp infection. The factors associated with the progression from *K. pneumoniae* colonization to infection are currently not well understood [5, 22]. The results of our study revealed that, although there was no microbiological profile related to the development of *K. pneumoniae* infection, diabetes mellitus was a potential risk factor for developing an infection (OR: 4.11; 95% CI: 1.14–14.8), in agreement with a previous report [22]. In a hvKp-rich cohort, as in our study, the virulence factor(s) can be offset or underestimated since hvKp-potential strains possessing gene clusters, such as virulence plasmid, are also detected in cKp and colonizers. Nevertheless, our results indicate that the K1 genotype, *rmpA*, and aerobactin are substantial predictors of hvKp infection. Each of these virulence factors may affect pathogenesis independently but in a coordinated manner; a recent study suggested that capsule biosynthesis, hypermucoviscosity, and metabolism coordinately affect *K. pneumoniae* fitness [23]. Conversely, a positive string test was inferior to these predictors and was less accurate for identifying hvKp (sensitivity of 84.2%, specificity of 43.3%, and positive LR of 1.49 [95% CI: 0.99–1.79]) than reported previously (sensitivity of 89% and specificity of 91%) [2]. As seen in our results, the inconsistency between *rmpA*/*rmpA2* and the hypermucoviscous phenotype has been found in 14% of 91 K*. pneumoniae* isolates in a previous report [24], which suggests that factors other than expolipopolysaccharide-associated genes (e.g., *magA* and *rmpA*/*rmpA2*) may be related to the expression of the hypermucoviscous phenotype. In addition to hypermucoviscosity, the overproduction of capsular polysaccharide (i.e., capsular polysaccharide thickness) can impact pathogenesis and biofilm formation, particularly in K1 serotype [23, 25]. Furthermore, experimental studies showed that aerobactin was the most critical hvKp-specific siderophore and enhanced pathogenesis, more so than yersiniabactin, salmochelin, or enterobactin [26, 27]. Thus, it is noteworthy that our study implies aerobactin may be a key virulence factor for hvKp, among several siderophores.

Third, to the best of our knowledge, this study is the first to indicate that the hvKp-related K1-ST82 cluster harbors distinct genomic backgrounds and virulence-gene profiles (compared with the K1-ST23 clone) by whole-genome sequencing. Previous genomic analyses showed that human clinical K1-ST23 isolates were clonal, and that colibactin, microcin E492, and yersiniabactin were K1-unique virulence factors [28, 29]. However, our findings revealed that virulence-gene profiles similar to that of K1-ST23 were also observed in K62-ST36, K20-ST268, K2-ST239, and K2-ST65, which implies that clinical features and severity are not always consistent with the molecular virulence potential. There is limited information on the clinical and molecular characteristics of K1-ST82. Data from a previous study showed that the pathogenic potential might differ between K1-ST82 and K1-ST23; however, the study included no hvKp-related K1-ST82 isolates [30]. In contrast, our results demonstrated that K1-ST82 was hypervirulent; four out of five (80%) isolates were hvKp. It is noteworthy that our data indicated that K1-ST82 strains with hypervirulent potential were distinct from the K1-ST23 clone in the population structure and that they harbored different virulence-gene profiles. Generally, MDR clones with antimicrobial-resistance genes and hypervirulent clones with virulence loci belong to different subsets [4], which is consistent with our findings. For example, two K21-ST323 isolates sharing a recent common ancestor with K1-ST23 were ESBL-producing strains and harbored few virulence genes. However, the findings of our study suggest an emerging issue, in that we identified a neighboring subclade of K1-ST23 comprising K54-ST29, K57-ST412, K62-ST36, and K20-ST268, which might have both MDR and hypervirulent potential [5, 31–33]. If so, this may be an alarming issue for clinicians since treatment of MDR hvKp infection can be challenging.

The present study has several limitations. First, this was a single-center study with a potential selection bias and only used univariate analyses; thus, it is uncertain whether the findings can be applied to other populations. However, the hvKp-rich population enabled us to design this study for a single center, and this study focused on *K. pneumoniae* taxonomy and the definition of hvKp provided above. As a result, we found that molecular factors (including the K1 genotype, *rmpA*, and aerobactin) could be key virulence factors for hvKp infection. We expect that prompt, simple, and accurate diagnostic tests will be developed to identify these factors and will be made available in clinical laboratories. Second, this study dose not closely emphasize on the phenotypic features of the strains, such as the biofilm formation, quantification of the capsular glucuronic acid for examining hypermucoviscous phenomenon [23, 34, 35], and resistance to macrophage-mediated phagocytosis. Nevertheless, it is noteworthy that our findings signify the huge impact of the genomic traits on the clinical virulence for tissue-invasive *K. pneumoniae* infection. Therefore, further investigations with precise bacteriological experiments are warranted to precisely evaluate the phenotypic features. Third, we studied the phylogenetic distributions of virulence genes in a relatively small population; thus, it was difficult to precisely define the relationships between clinical severity and virulence genes. This is because the virulence genes analyzed in this study are not part of the core *K. pneumoniae* genome, but are accessory genes [22], leading to virulence-gene profiles that do not affect the overall population structure. Further international collaboration is warranted to elucidate vital virulence factors in *K. pneumoniae* and to analyze strains linked to more clinical information.

## Conclusions

The present findings showed that, particularly in an hvKp-endemic area, clinical characteristics including diabetes mellitus and community-acquired infection are no longer useful for predicting hvKp infection, whereas the K1 genotype, *rmpA*, and aerobactin are meaningful predictors of hvKp. We believe that these predictors will be useful for decision making regarding whether to additionally examine pyogenic (metastatic) infection caused by *K. pneumoniae* in clinical practice in the future. We expect that our novel findings involving the characteristics of K1-ST82 and the presence of a clade carrying MDR and hypervirulent potential can promote the further understanding of antimicrobial resistance and hypervirulence in *K. pneumoniae*.

## Supplementary Information

**Additional file 1: Table S1.** Data set of 112 *Klebsiella pneumoniae* isolates in AIH, Japan for genomic analyses.

**Additional file 2: Table S2.** Clinical and microbiological characteristics of the patients infected with *K. pneumoniae* species complex (n = 16). **Table S3.** Clinical and microbiological characteristics of the patients: infected versus colonized (n = 112). **Table S4.** Variables analyzed for the prediction of *K. pneumoniae* infection. **Table S5.** Clinical and microbiological characteristics of patients with hvKp infection (n = 19).

## Data Availability

The nucleotide-sequence data presented here were deposited in the DNA Data Bank of Japan Sequenced Read Archive under accession numbers DRX270567-270695 (http://www.ddbj.nig.ac.jp/index-e.html). Other data that support the findings of this study are available from the corresponding author upon reasonable request.

## Abbreviations

AIH: Aso Iizuka Hospital
cKp: Classical *K. pneumonia*
CI: Confidence interval
ESBL: Extended-spectrum beta-lactamase
hvKp: Hypervirulent *K. pneumonia*
ICU: Intensive care unit
LR: Likelihood ratio
MALDI–TOF MS: Matrix-assisted laser desorption/ionization–time-of-flight mass spectrometry
MDR: Multidrug resistant
OR: Odds ratio
ST: Sequence type
SNVs: Single-nucleotide variations
