# Supplementary material for: Genomic insights into virulence factors affecting tissue-invasive Klebsiella pneumoniae infection
Source: Ann Clin Microbiol Antimicrob. 2022 Feb 5;21:2. doi: 10.1186/s12941-022-00494-7 (PMC8817621; doi:10.1186/s12941-022-00494-7)
Supplement: Supplementary file 2 — Additional file 2: Table S2. Clinical and microbiological characteristics of the patients infected with K. pneumoniae species complex (n = 16). Table S3. Clinical and microbiological characteristics of the patients: infected versus colonized (n = 112). Table S4. Variables analyzed for the prediction of K. pneumoniae infection. Table S5. Clinical and microbiological characteristics of patients with hvKp infection (n = 19). [file 12941_2022_494_MOESM2_ESM.docx]

| Table S2. Clinical and microbiological characteristics of the patients infected with *K. pneumoniae* species complex (n = 16) | | | | | | | |  |  |  |  |  |  |  |
| --- | --- | --- | --- | --- | --- | --- | --- | --- | --- | --- | --- | --- | --- | --- |
| Strain no. | Strain | Age | Community-acquired | Charlson index | Site(s) of infection | Bacteremia | In-hospital death | String test | Aerobactin | Yersiniabactin | Salmochelin | Colibactin | *rmpA* | *rmpA2* |
| KP19011AIH | *K. africana* | 73 | + | 1 | Cholangitis | – | – | – | – | – | – | – | – | – |
| KP17007AIH | *K. variicola* subsp. *variicola* | 79 | + | 1 | Cholecystitis | + | – | + | – | – | – | – | – | – |
| KP18030AIH | *K. variicola* subsp. *variicola* | 97 | + | 5 | None (carriage) | – | – | + | – | – | – | – | – | – |
| KP18032AIH | *K. variicola* subsp. *variicola* | 79 | + | 8 | Pyelonephritis | – | – | – | – | + | – | – | – | – |
| KP18033AIH | *K. variicola* subsp. *variicola* | 64 | + | 2 | Pyelonephritis | – | – | – | – | – | – | – | – | – |
| KP18034AIH | *K. variicola* subsp*. variicola* | 68 | + | 2 | Cholangitis | + | – | – | – | – | – | – | – | – |
| KP18040AIH | *K. variicola* subsp. *variicola* | 64 | – | 5 | Cholangitis | + | – | – | – | – | – | – | – | – |
| KP18072AIH | *K. quasipneumoniae* subsp. *quasipneumoniae* | 95 | + | 4 | Cholangitis | – | – | – | – | – | – | – | – | – |
| KP19020AIH | *K. quasipneumoniae* subsp. *quasipneumoniae* | 68 | + | 2 | Cholangitis | + | – | – | – | – | – | – | – | – |
| KP17003AIH | *K. quasipneumoniae* subsp*. similipneumoniae* | 101 | – | 2 | Cholangitis | – | – | + | – | – | – | – | – | – |
| KP18037AIH | *K. quasipneumoniae* subsp. *similipneumoniae* | 67 | + | 0 | Pneumonia | – | – | – | – | – | – | – | – | – |
| KP18049AIH | *K. quasipneumoniae* subsp. *similipneumoniae* | 76 | – | 9 | Pyelonephritis | – | – | – | – | – | – | – | – | – |
| KP18051AIH | *K. quasipneumoniae* subsp. *similipneumoniae* | 68 | + | 2 | Pyelonephritis | – | – | – | – | – | – | – | – | – |
| KP18063AIH | *K. quasipneumoniae* subsp. *similipneumoniae* | 69 | + | 2 | Cholangitis | + | – | – | – | – | – | – | – | – |
| KP18074AIH | *K. quasipneumoniae* subsp. *similipneumoniae* | 84 | + | 3 | Pyelonephritis | – | – | + | – | – | – | – | – | – |
| KP19014AIH | *K. quasipneumoniae* subsp. *similipneumoniae* | 88 | – | 3 | Pyelonephritis, Pneumonia | – | – | – | – | – | – | – | – | – |

| Table S3. Clinical and microbiological characteristics of the patients: infected *versus* colonized (n = 112) | | | |
| --- | --- | --- | --- |
|  | Infection (n = 86) | Colonization (n = 26) | p value |
| Age (years), median (IQR) | 75 (69–84) | 74 (65–83) | 0.26 |
| Male, n (%) | 52 (60) | 17 (65) | 0.65 |
| Comorbidity, n (%) |  |  |  |
| Diabetes mellitus | 30 (35) | 3 (12) | 0.022 |
| Malignancy | 24 (28) | 8 (31) | 0.78 |
| Immunocompromised conditions | 11 (13) | 2 (8) | 0.38 |
| Liver cirrhosis | 4 (5) | 1 (4) | 0.67 |
| Chronic kidney disease | 8 (9) | 4 (15) | 0.29 |
| Charlson index, median (IQR) | 2 (2–4) | 2 (1–3) | 0.26 |
| Microbiological profile, n (%) |  |  |  |
| Positive string test | 54 (64) | 22 (85) | 0.037 |
| O1^*^ | 53 (62) | 13 (50) | 0.29 |
| O2^*^ | 22 (26) | 10 (38) | 0.22 |
| O3 | 9 (10) | 3 (12) | 0.56 |
| K1 | 13 (15) | 4 (15) | 0.6 |
| K2 | 28 (33) | 7 (27) | 0.66 |
| Aerobactin | 52 (60) | 19 (73) | 0.24 |
| Yersiniabactin | 42 (49) | 18 (69) | 0.068 |
| Salmochelin | 49 (57) | 19 (73) | 0.14 |
| Colibactin | 23 (27) | 12 (46) | 0.061 |
| *rmpA* | 51 (59) | 17 (65) | 0.58 |
| *rmpA2* | 42 (49) | 17 (65) | 0.14 |
| IQR, interquartile range |  |  |  |
| *Three O1/O2v2 strains involved in infection and one in colonization | | | |

| Table S4. Variables analyzed for the prediction of *K. pneumoniae* infection | | |
| --- | --- | --- |
| Variables | OR (95% CI) | p value |
| Diabetes mellitus | 4.11 (1.14–14.8) | 0.031 |
| Positive string test | 0.31 (0.097–0.97) | 0.044 |
| O1 | 1.61 (0.66–3.88) | 0.29 |
| O2 | 0.55 (0.22–1.39) | 0.55 |
| K1 | 0.98 (0.29–3.31) | 0.97 |
| K2 | 1.24 (0.47–3.31) | 0.66 |
| Aerobactin | 0.56 (0.21–1.48) | 0.25 |
| Yersiniabactin | 0.42 (0.17–1.08) | 0.072 |
| Salmochelin | 0.49 (0.19–1.28) | 0.15 |
| Colibactin | 0.43 (0.17–1.06) | 0.065 |
| *rmpA* | 0.31 (0.31–1.93) | 0.58 |
| *rmpA2* | 0.51 (0.20–1.26) | 0.14 |
| OR, odds ratio; CI, confidence interval | |  |

| Table S5. Clinical and microbiological characteristics of patients with hvKp infection (n = 19) | | | | | | | | | |  |  |  |  |  |  |  |
| --- | --- | --- | --- | --- | --- | --- | --- | --- | --- | --- | --- | --- | --- | --- | --- | --- |
| Strain no. | Age | Community-acquired | Charlson index | Site(s) of infection | Bacteremia | In-hospital death | String test | Capsular genotype | LPS genotype | ST | Aerobactin | Yersiniabactin | Salmochelin | Colibactin | *rmpA* | *rmpA2* |
| KP18005AIH | 74 | + | 1 | Liver abscess | + | – | + | K1 | O1v2 | 23 | + | + | + | + | + | + |
| KP18054AIH | 64 | – | 2 | Lung abscess, empyema | + | + | + | K1 | O1v2 | 23 | + | + | + | + | – | – |
| KP18066AIH | 74 | + | 1 | Liver abscess | – | – | + | K1 | O1v2 | 23 | + | + | + | + | + | + |
| KP19005AIH | 69 | + | 1 | Liver abscess, intraperitoneal abscess | + | – | + | K1 | O1v2 | 23 | + | + | + | + | + | + |
| KP17009AIH | 60 | + | 0 | Ovarian abscess | – | – | + | K1 | O1v1 | 82 | + | – | – | – | + | – |
| KP18003AIH | 69 | – | 1 | Pneumonia, empyema | + | – | + | K1 | O1v1 | 82 | + | – | – | – | + | – |
| KP18062AIH | 59 | + | 0 | Lung abscess | + | – | + | K1 | O1v1 | 82 | + | – | – | – | + | – |
| KP19008AIH | 74 | – | 2 | Pneumonia, lung abscess | + | + | + | K1 | O1v1 | 82 | + | – | – | – | + | – |
| KP17025AIH | 50 | + | 0 | Appendicitis, peritonitis, intraperitoneal abscess | – | – | + | K2 | O1v2 | 65 | + | + | + | + | + | + |
| KP18065AIH | 90 | – | 3 | Empyema | + | + | – | K2 | O1v2 | 65 | + | – | + | – | + | + |
| KP19001AIH | 76 | + | 0 | Pneumonia, lung abscess | + | + | + | K2 | O1v2 | 65 | + | – | + | – | + | + |
| KP18001AIH | 74 | – | 4 | Meningitis | + | – | + | K2 | O1v1 | 86 | + | + | + | – | + | + |
| KP18012AIH | 82 | – | 7 | Lung abscess | – | + | + | K2 | O1v1 | 86 | + | + | + | – | + | + |
| KP19007AIH | 69 | + | 4 | Liver abscess, pulmonary abscess | + | – | + | K2 | O1v1 | 2039 | + | + | + | + | + | – |
| KP18016AIH | 67 | + | 1 | Liver abscess | + | – | + | K54 | O1v2 | 29 | + | + | + | – | + | + |
| KP19004AIH | 75 | + | 3 | Liver abscess | + | – | – | K54 | O1v2 | 29 | – | + | + | – | + | – |
| KP19002AIH | 90 | – | 3 | Liver abscess, intraperitoneal abscess | + | – | – | K58 | O3b | 1017 | – | – | – | – | – | – |
| KP19023AIH | 69 | – | 2 | Lung abscess | + | + | + | K62 | O2v1 | 36 | + | + | + | + | + | + |
| KP17010AIH | 75 | + | 4 | Empyema | – | – | + | K108 | O1v2 | 35 | – | + | + | – | + | – |
| hvKp, hypervirulent *K. pneumoniae*; LPS, lipopolysaccharide; ST, sequence type | | | | | | | | |  |  |  |  |  |  |  |  |
